# Anti-Fine Dust Effect of Fucoidan Extracted from *Ecklonia maxima* Laves in Macrophages via Inhibiting Inflammatory Signaling Pathways

**DOI:** 10.3390/md20070413

**Published:** 2022-06-24

**Authors:** D.P. Nagahawatta, N.M. Liyanage, H.H.A.C.K. Jayawardhana, Hyo-Geun Lee, Thilina U. Jayawardena, You-Jin Jeon

**Affiliations:** 1 Department of Marine Life Sciences, Jeju National University, Jeju 690-756, Korea; pramuditha1992@jejunu.ac.kr (D.P.N.); liyanagenm@jejunu.ac.kr (N.M.L.); chathuri.k.j@office.jejunu.ac.kr (H.H.A.C.K.J.); hyugeunlee92@jejunu.ac.kr (H.-G.L.); 2 Department of Cell Biology & Anatomy, Arnie Charbonneau Cancer and Alberta Children’s Hospital Research Institutes, Cumming School of Medicine, University of Calgary, Calgary, AB T2N 1N4, Canada; 3 Marine Science Institute, Jeju National University, Jeju 63333, Korea

**Keywords:** fucoidan, anti-fine dust, *Ecklonia maxima*, anti-inflammation, NF-κB, MAPK, TLR, signaling pathways

## Abstract

Brown seaweeds contain fucoidan, which has numerous biological activities. Here, the anti-fine-dust activity of fucoidan extracted from *Ecklonia maxima*, an abundant brown seaweed from South Africa, was explored. Fourier transmittance infrared spectroscopy, high-performance anion-exchange chromatography with pulsed amperometric detection analysis of the monosaccharide content, and nuclear magnetic resonance were used for the structural characterization of the polysaccharides. The toll-like receptor (TLR)-mediated nuclear factor kappa B (NF-κB) and mitogen-activated protein kinase (MAPK) signaling pathways were evaluated. The results revealed that *E. maxima* purified leaf fucoidan fraction 7 (EMLF7), which contained the highest sulfate content, showed the best anti-inflammatory activity by attenuating the TLR-mediated NF-κB/MAPK protein expressions in the particulate matter-stimulated cells. This was solidified by the successful reduction of Prostaglandin E_2_, NO, and pro-inflammatory cytokines, such as TNF-α, IL-6, and IL-1β. The current findings confirm the anti-inflammatory activity of EMLF7, as well as the potential use of *E. maxima* as a low-cost fucoidan source due to its abundance. This suggests its further application as a functional ingredient in consumer products.

## 1. Introduction

Urban air pollution caused by particulate matter (PM) composed of solid and liquid particles of different sizes and origins has become a major threat to civilization [1]. The high amount of PM in the environment has led to various environmental issues as well as human health implications leading to mortality. It has been identified that exposure to PM is correlated with increased risk of pulmonary diseases, such as asthma, cancer, etc. [2]. The World Health Organization reported that PM contributes to an increased number of premature deaths annually [3]. PM consists of numerous substances, including molds, pollens, mineral dust, and microorganisms. Among these, aromatic hydrocarbons, heavy metals, and nitrate compounds are well known for stimulating allergic and inflammatory responses in cells [4,5]. Numerous studies have reported on the ability of PM to induce inflammation, which leads to deleterious effects on the human body [6,7,8,9,10]. Therefore, it is important to investigate possible treatments to counteract PM-induced diseases.

Inflammation is a host’s defensive mechanism response to tissue injuries, stress, or oxidative stress. Excessive and continuous inflammatory responses can have a negative impact on the host, leading to tissue damage [11,12]. Rheumatoid arthritis, inflammatory bowel disease, Alzheimer’s disease, and cardiovascular diseases are some conditions in which pathogenesis (chronic inflammation) is involved [13]. Inflammation is characterized by excessive production of cytokines or acute-phase reactants, such as reactive oxygen species (ROS), which activate inflammatory signaling pathways. The downregulation of pro-inflammatory factors is considered a viable therapeutic approach for curing inflammatory diseases. 

Fucoidan is a sulfated polysaccharide unique to brown algae, which possesses a broad range of bioactive properties. Fucose and sulfate groups are its main components, while galactose, xylose, mannose, and uronic acids are its auxiliary components that have garnered significant attention. Fucoidans have a high degree of molecular structural diversity, which depends on the specificity of the species, inter-compositional variation between several species, and intra-compositional variation between the same species from which they are harvested. These structural features play a major role in their biological activities [14]. This polysaccharide has a wide range of biological activities, such as anti-oxidant, anti-coagulant, anti-inflammatory, anti-viral, and anti-diabetic properties [15]. In recent years, it was demonstrated that fucoidan exhibited antiviral activities both in vivo and in vitro due to its low cytotoxicity compared with other antiviral drugs currently available in clinical medicine. Fucoidan from *Undaria pinnatifida* and *Cystoseira indica* has been proven to have antiviral activity against Herpes infection [16]. Furthermore, it has been proven that fucoidan can inhibit the replication of several enveloped viruses, such as human immunodeficiency and human cytomegalovirus [17]. Many studies have shown that fucoidan possesses remarkable antioxidant activity and is a natural antioxidant that prevents free-radical-mediated diseases [18]. This antioxidant activity was reported to be dependent on its molecular weight and sulfate content. Apart from the mentioned bioactivities, fucoidans isolated from several seaweed species are considered good anticoagulant agents due to their effective thrombin and factor Xa inhibitory activity [19]. The modulating ability of marine fucoidans as anti-inflammatory agents toward inflammatory mediators, such as nitric oxide (NO), Prostaglandin E2 (PGE2), and pro-inflammatory cytokines, has been previously demonstrated [20,21,22,23]. Fucoidan from several seaweeds has been proven to act as a ligand for macrophage scavenger receptors and inhibit NO production. Moreover, this sulfate polysaccharide isolated from brown algae has been shown to inhibit the migration of leukocytes to inflammatory tissues manifesting anti-inflammatory activity [24]. Fucoidan isolated from brown algae has been tested for its ability to enhance the efficacy of anti-inflammatory drugs. Several previous studies have reported the anti-inflammatory activity of fucoidan in different experimental models, including in vitro and in vivo, such as developing an anti-inflammatory lotion using fucoidan isolated from *Fucus vesiculosus* [25,26], anti-colon-tumor effects, anti-breast-cancer effects, and anti-lung-cancer effect [27]. However, the anti-inflammatory activity of fucoidan isolated from *Ecklonia maxima* has not been extensively studied. 

*E. maxima* is a brown algal species predominantly found in South African coastal areas that are considered to be highly biodiverse, with a number of untapped pristine marine protected areas. Globally, South Africa ranks third in terms of its rich biodiversity, where 80% of the flora is unique to the country [28]. In 2014, more than 12,000 metric tons of fresh *E. maxima* were harvested; it is primarily used as a feed for abalone farms [29]. The seaweed is also processed into plant-growth stimulants, which are marketed globally. Owing to its high abundance, it can be used as an inexpensive source for extracting bioactive compounds for their utilization in various consumer products. Like other brown algal species, *E. maxima* has anti-diabetes, anti-cancer, and anti-oxidant activities [30,31]. However, to the best of our knowledge, the anti-inflammatory mechanism of fucoidan isolated from *E. maxima* has not been systematically investigated. Therefore, the present study focuses on the effect of fucoidan from *E. maxima* as a therapeutic agent against PM-induced inflammation in RAW 264.7 cells.

## 2. Results

### 2.1. Proximate Chemical Composition of E. Maxima and its Polysaccharide Fractions

**Figure 1:**
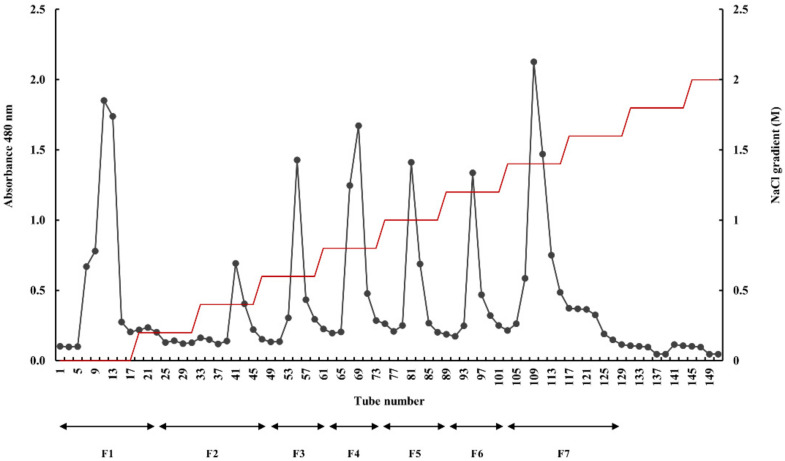
Purification through DEAE–cellulose anion exchange chromatography to obtain the fractions of fucoidan.

The chemical compositions of the crude extract, Celluclast-assisted extract, and the ethanol-precipitated component of *E. maxima* were determined. The proximate composition results were reported in our previous study [32]. The polysaccharide and sulfate contents of the Celluclast-assisted extract were 42.37 ± 0.48% and 6.29 ± 0.56%, respectively, while those of the ethanol-precipitated component were 69.37 ± 0.16% and 10.51 ± 0.23%, respectively. The crude polysaccharide fraction was then purified into seven fractions using a diethylaminoethyl (DEAE)–cellulose column (Figure 1). A proximate compositional analysis of each fraction was performed to determine the polysaccharide, protein, sulfate, and polyphenol contents (Table 1), which showed that *E. maxima* leaf fucoidan fraction (EMLF) 7 had the highest sulfate content.

### 2.2. Fourier Transform Infrared (FTIR) Spectra and Molecular Weight Determination of the Isolated Fucoidan Fractions

**Figure 2:**
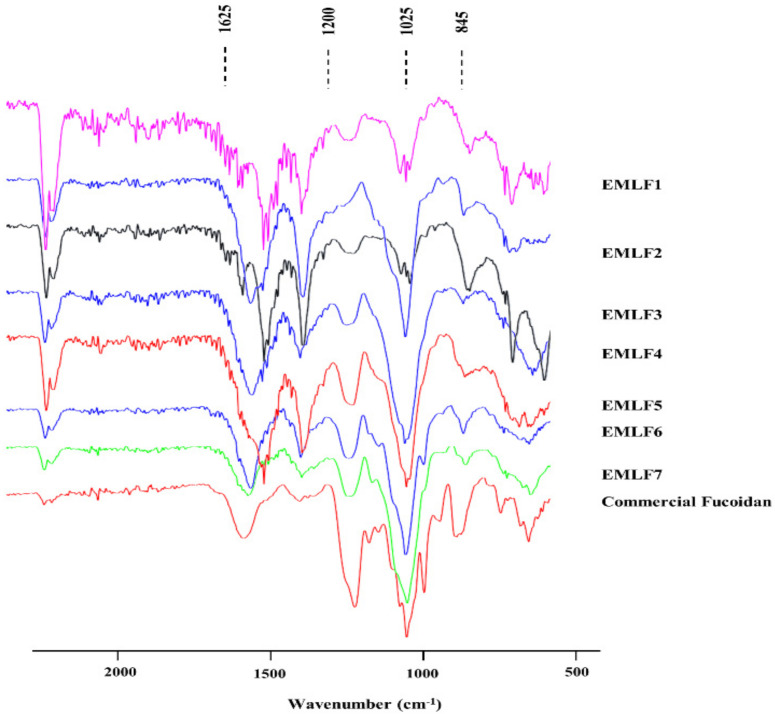
FTIR chromatography. Purified *E. maxima* leaf Fucoidan (EML) FTIR analysis.

The FTIR spectra of commercial fucoidan and the fucoidan fractions extracted from *E. maxima* were within the range of 2000–500 cm^−1^ (Figure 2). The prominent IR band at 1025 cm^−1^ denoted the C-O-C stretching vibration in commercial fucoidan and EMLF7. S = O stretching vibrations were represented by the bands at 1245–1255 cm^−1^, indicating a significant amount of sulfate via strong absorption. The sharp peak at 845 cm^−1^ and the shoulder peak at 825 cm^−1^ denoted the substitution of the sulfate group at the C-4 position (C-O-S). The traceable moisture content of the sample was indicated by the bending vibrations of H-O-H in the 1600 cm^−1^ region. The peak at 1625 cm^−1^ represented the C = O stretching vibration of the carboxylic ester [33,34,35,36]. Further, there were differences in the relative expressions of the fractions, while EMLF7 was the most similar to the commercial fucoidan fraction. The molecular weight determination results confirmed the homogeneous molecular weight of each fraction, successful separation of polysaccharide fractions, and gradual decrease in the molecular weight with the fraction number (Supplementary Figure S1).

### 2.3. Nuclear Magnetic Resonance Spectroscopy (NMR) and Monosaccharide Composition of the Active Fraction

**Figure 3:**
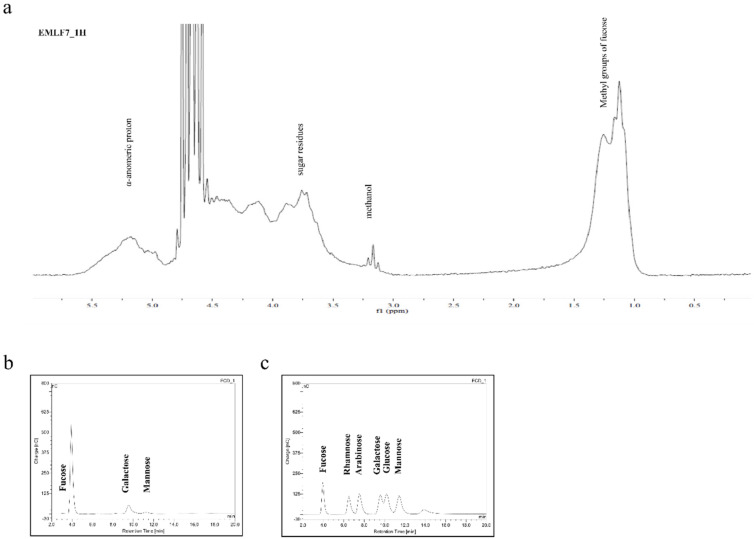
The 1H NMR and monosaccharide analysis of *E. maxima* purified leaf fucoidan fraction 7 (EMLF7). (**a**) The 1H NMR spectrum of EMLF7; (**b**) monosaccharide content of EMLF7 analyzed by the HPAE-PAD spectrum compared with (**c**) a standard monosaccharide mixture.

The NMR spectrum of EMLF7 is shown in Figure 3a. Intense signals in the range of 5–5.5 ppm were indicative of the α-anomeric protons (H1 of α-L-fucopyranose). The high field signal of 1.10 ppm denoted the C6 methyl protons of L-fucose. Sugar residue protons were indicated by the peaks in the range of 3.4–4.0 ppm. The observed peaks and the corresponding chemical characteristics verified EMLF7 as fucoidan. However, the 13C NMR spectra were not satisfactory because of the heterogeneity and complexity of the polysaccharide structure of fucoidan [37,38,39,40].

The monosaccharide composition of each fraction (Table 2) was recorded using a high-performance anion exchange chromatography with pulsed amperometric detection (HPAE-PAD) system (Dionex, Sunnyvale, California, USA) and compared with a standard monosaccharide mixture (Figure 3b,c). Compared with the other fractions, EMLF7 contained the highest amount of fucose (81.83%).

### 2.4. Fucoidan Extracted from E. Maxima Inhibited NO Production in PM-Stimulated RAW 264.7 Cells

**Figure 4:**
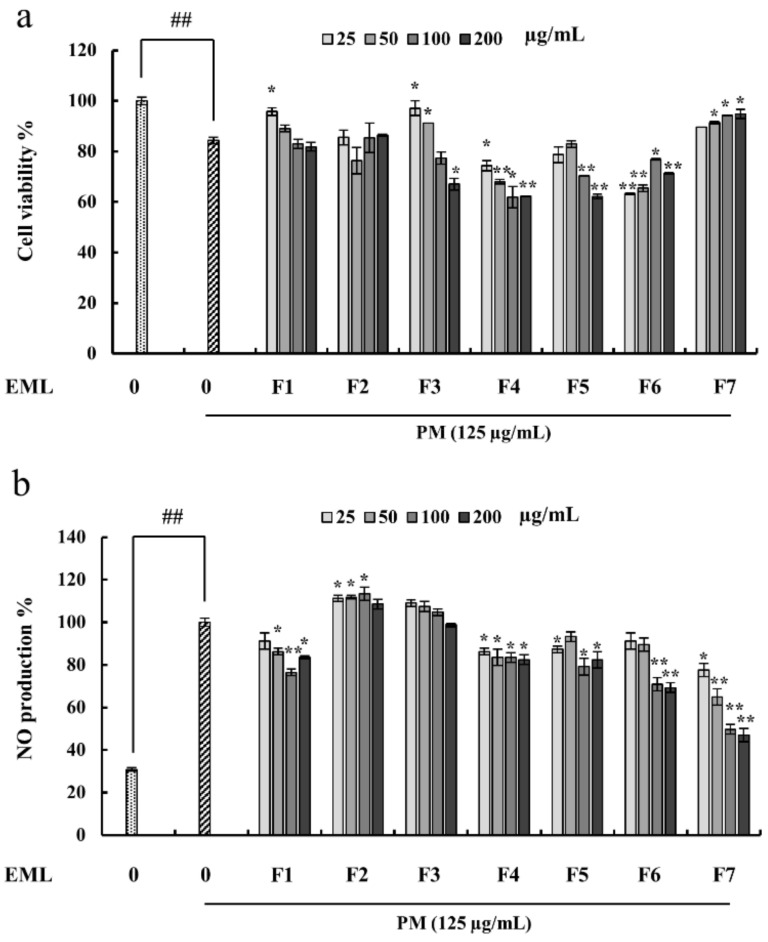
Protective effect of *E. maxima* purified leaf fucoidan (EML) fraction 1–7 (F1-F7) against particulate matter (PM)-induced (**a**) toxicity and (**b**) NO production and in RAW 264.7 cells. Experiments were carried out in triplicate and the results are represented as means ± SD (n = 3). Values are significantly different from the PM-treated group at * *p* < 0.05 and ** *p* < 0.01 or ## *p* < 0.01 against the control.

In this study, RAW 264.7 cells were pretreated with the seven fucoidan fractions prior to PM stimulation. The cell viability of the untreated PM-stimulated RAW 264.7 cells drastically decreased (Figure 4a) compared with the treated group. Furthermore, PM stimulation increased NO production in the RAW 264.7 cells, which was significantly inhibited by the treatment with the fucoidan extracts, depending on the dose (Figure 4b). Compared with the other fractions, EMLF7 had superior cytoprotective and NO suppressive effects (Figure 4). Therefore, EMLF7 was used for the subsequent experiments.

### 2.5. EMLF7 Attenuated PGE2 and Pro-Inflammatory Cytokine Secretion in PM-Stimulated RAW 264.7 Cells

**Figure 5:**
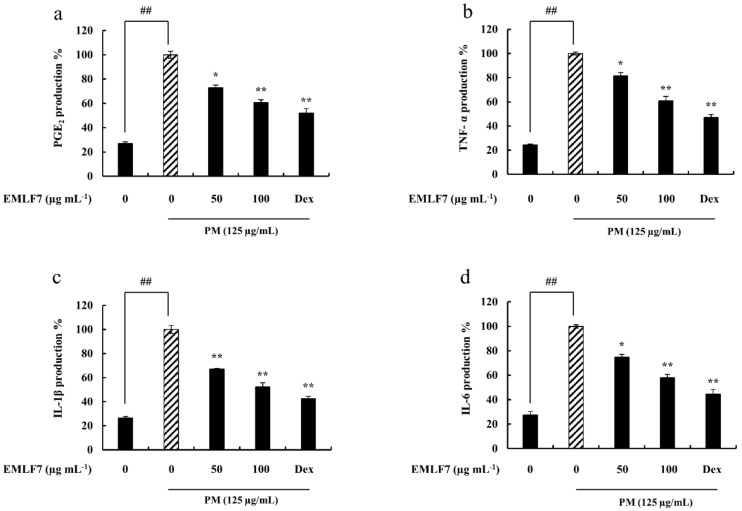
*E. maxima* purified leaf fucoidan fraction 7 (EMLF7) inhibited the particulate matter (PM)- induced production of (**a**) PGE2, (**b**)TNF-α, (**c**) IL-1β, and (**d**) IL-6 pro-inflammatory cytokines. Experiments were carried out in triplicate and the results are represented as means ± SD (n = 3). Values are significantly different from the PM-treated group at * *p* < 0.05 and ** *p* < 0.01 or ## *p* < 0.01 against control. An amount of 50 µM of Dexamethasone (Dex) was used as a positive control.

PGE2 is an important inflammatory mediator, and the overproduction of pro-inflammatory cytokines, such as IL-6, IL-1β, and TNF-α, can significantly influence pathogenesis in various inflammatory diseases [41]. Therefore, to determine the effects of EMLF7 on their production in PM-stimulated RAW 264.7 cells, an enzyme-linked immunosorbent assay (ELISA) assay was conducted. The releases of PGE2 and pro-inflammatory cytokine were significantly higher in the PM-stimulated macrophage cells. On the other hand, EMLF7 inhibited the PM-mediated production of PGE2 and the other cytokines in a dose-dependent manner (50 and 100 μg/mL). Dexamethasone was used as a positive control in this experiment (Figure 5).

### 2.6. EMLF7 Reduced the Expression Levels of iNOS and COX-2 Proteins in PM-Stimulated Macrophage Cells

**Figure 6:**
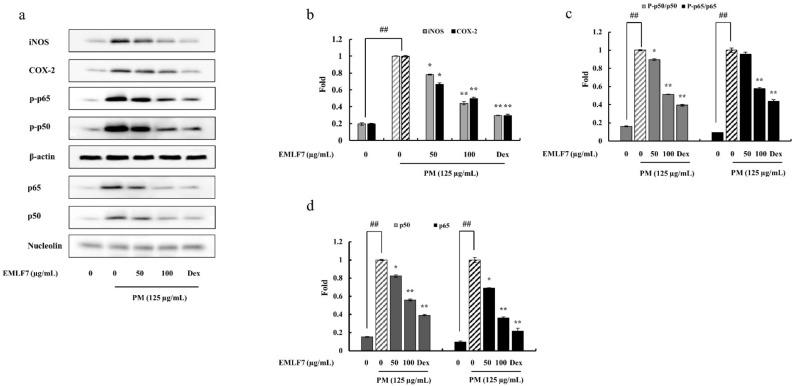
Effects of the *E. maxima* purified leaf fucoidan fraction 7 (EMLF7) on particulate matter (PM)-induced iNOS, COX2, and NF-κB pathway protein expression in RAW 264.7 cells. (**a**) Expression analysis of iNOS, COX2, p50, p65, and phosphorylation in cytosol and nucleus evaluated using Western blotting after the treatment of EMLF7 in PM-activated macrophages, (**b**) quantification of iNOS and COX-2 expression, (**c**) quantification of p50 and p65 in cytosol, and (**d**) quantification of p50 and p65 in the nucleus. Experiments were carried out in triplicate and the results are represented as means ± SD (n = 3). Values are significantly different from the PM-treated group at * *p* < 0.05 and ** *p* < 0.01 or ## *p* < 0.01 against the control. An amount of 50 µM of Dexamethasone (Dex) was used as a positive control.

Western blot analysis was used to evaluate the inhibitory effect of EMLF7 on iNOS and COX-2 proteins in PM-stimulated macrophage cells (Figure 6a,b). The PM stimulation significantly increased the iNOS and COX-2 levels in the RAW 264.7 cells, which was considered to cause the upregulation of NO and PGE2 in the stimulated cells. However, depending on the dose, EMLF7 successfully suppressed the iNOS and COX-2 levels.

### 2.7. Inhibition of the NF-κB Phosphorylation and MAPK Signaling Pathways by EMLF7

**Figure 7:**
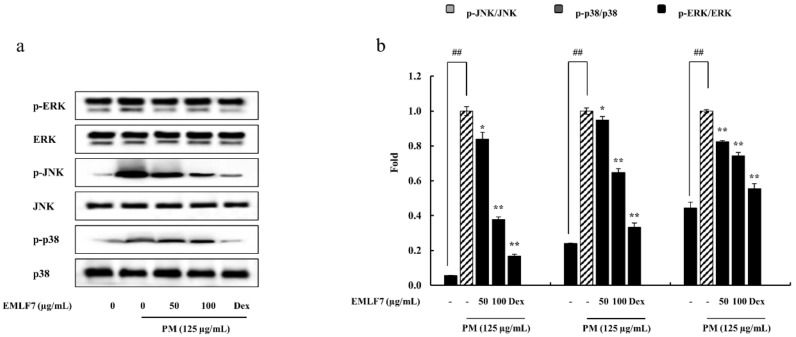
The effects of the *E. maxima* purified leaf fucoidan fraction 7 (EMLF7) on the particulate matter (PM)-induced phosphorylation and translocation of MAPK-related protein expressions. (**a**) Protein expression analysis of p-ERK, ERK, p-JNK, JNK, p-p38, and p38, and (**b**) quantification of these protein expressions. Experiments were carried out in triplicate and the results are represented as means ± SD (n = 3). Values are significantly different from the PM-treated group at * *p* < 0.05 and ** *p* < 0.01 or ## *p* < 0.01 against the control. An amount of 50 µM of Dexamethasone (Dex) was used as a positive control.

The present study evaluated the expression and translocation of p50, p65, ERK, JNK, and p38 in EMLF7-treated PM-stimulated cells. The phosphorylation levels of NF-κB, p50, and p65 in the cytosol increased with PM activation (Figure 6c) as compared with the control cells (no PM exposure). Likewise, the nucleus translocation of p50 and p65 was upregulated by PM activation, as expected (Figure 6d). However, the EMLF7 treatment (50 and 100 μg/mL) prior to PM stimulation significantly reduced the phosphorylation and nuclear translocation of p50 and p65. In addition, the effect of EMLF7 on the MAPK signaling pathway was tested by Western blot analysis. Similar to the NF-κB pathway proteins, PM stimulation led to the significant activation of p38, ERK, and JNK, which were successfully downregulated by EMLF7 (Figure 7). 

### 2.8. Downregulation of the iNOS and COX-2 Gene Expressions, and Pro-Inflammatory Cytokine mRNA Expression in PM-Stimulated RAW 264.7 Cells by EMLF7

**Figure 8:**
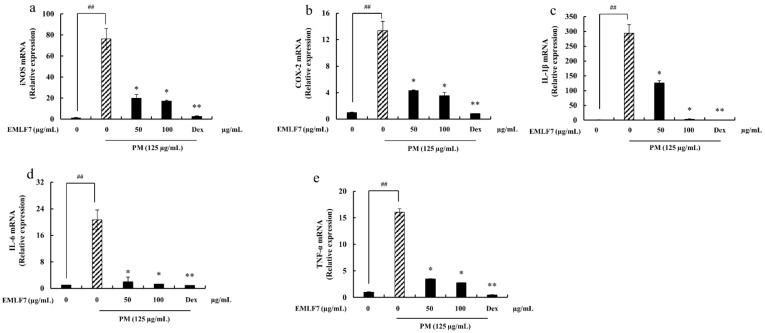
Effect of *E. maxima* purified leaf fucoidan fraction 7 (EMLF7) on iNOS, COX-2, TNF-α, IL-6, and IL-1β mRNA expression in PM-induced RAW 264.7 macrophages. The mRNA expression levels of (**a**) iNOS, (**b**) COX-2, (**c**) IL-1 β, (**d**) IL-6, and (**e**) TNF-α were quantitated with real-time fluorescence quantitative PCR. Experiments were carried out in triplicate and the results are represented as means ± SD (n = 3). Values are significantly different from the PM-treated group at * *p* < 0.05 and ** *p* < 0.01 or ## *p* < 0.01 against the control. An amount of 50 µM of Dexamethasone (Dex) was used as a positive control.

In addition to the PM-induced iNOS and COX-2 protein level expressions, the gene expression levels were also studied using RT-qPCR (Figure 8a,b). The results indicated that the mRNA production of iNOS and COX-2 was elevated in response to PM, while it was significantly lowered upon treatment with EMLF7. A drastic increase in the pro-inflammatory cytokine (IL-6, IL-1β, and TNF-α) mRNA production was also observed in the PM-stimulated RAW 264.7 cells (Figure 8c–e), while the EMLF7 treatment (50–100 μg/mL) efficiently regulated the IL-6, IL-1β, and TNF-α gene expression. 

### 2.9. Inhibition of PM-Stimulated Toll-Like Receptor (TLR)-2 and TLR-4 mRNA Expressions by the EMLF7 Treatment in RAW 264.7 Cells

**Figure 9:**
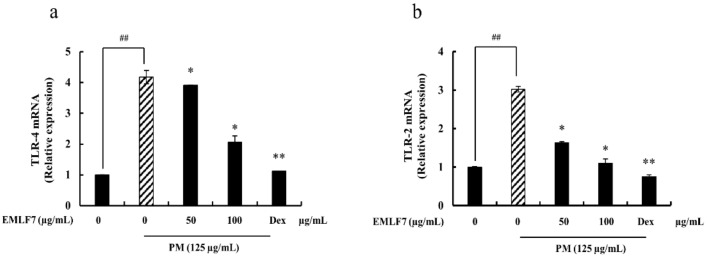
Effect of *E. maxima* purified leaf fucoidan fraction 7 (EMLF7) on TLR mRNA expression in PM-induced RAW 264.7 macrophages. Experiments were carried out in triplicate and the results are represented as means ± SD (n = 3). Values are significantly different from the PM-treated group (**a**) at * *p* < 0.05 and ** *p* < 0.01 or ## *p* < 0.01 against the control. (**b**) An amount of 50 µM of Dexamethasone (Dex) was used as a positive control.

TLRs (TLR-2 and TLR-4) play a major role in the innate immune system by sensing initial infections. In addition, they are the most potent inducers of the inflammatory response [34]. Hence, the effect of EMLF7 on these receptors was tested with qPCR. When stimulated with PM, the mRNA production of TLR-2 and TLR-4 was significantly increased compared with the control group (no PM exposure), while their levels were considerably downregulated by EMLF7 (Figure 9). These results strongly suggest that EMLF7 downregulated the inflammatory responses caused by PM stimulation in the RAW 264.7 macrophage cells.

## 3. Discussion

Air pollutants may include any toxic gases or airborne particles with an aerodynamic diameter smaller than 10 μm. These fine particles are composed of both organic and inorganic compounds, or just one type. The size, composition, and source of origin of these particles are based on their microenvironment. PM has been a serious threat, especially in East Asian countries, such as China, Korea, and Japan [42,43,44], by generating various inflammatory diseases in several parts of the body, such as the skin, lungs, and the heart. PM inhalation may induce allergic inflammation, while the penetration of the skin keratinocytes results in oxidative stress and inflammation [45]. Fucoidan is a sulfated polysaccharide unique to brown algae that possesses a broad range of bioactive properties. Owing to its anti-inflammatory properties, fucoidan can be used as an anti-inflammatory agent [33,46,47,48,49,50]. The present study was conducted to demonstrate and evaluate the anti-inflammatory activity of *E. maxima* leaf fucoidan against PM-induced inflammation in vitro. 

The method used in this study to extract fucoidan from seaweed increased the extraction efficacy and purity of the final product [50]. Brown algae contain various pigments, such as xanthophyll and fucoxanthin, and polyphenols, such as phlorotannins [51]. These interfering compounds were removed from the sample using 10% formaldehyde in ethanol. The samples were also depigmented and de-fatted using ethanol. The purification procedure facilitated the formation of the phenolic polymer, resulting in the lower solubility of phenolic substances. Celluclast, as a catalyst, increases the rate of the reactions that convert the substrate into products. It breaks down the glucose polymer into glucose and longer-chained units. The reaction is maximized at pH 4.5 and 50 °C; these conditions also conserve the bioactivity of the extracted fucoidan [52]. 

The seven fractions were obtained by elution from the DEAE–cellulose column, namely EMLF1–EMLF7, which were freeze-dried and dialyzed. We previously reported on the composition of the seven fucoidan fractions while determining their antioxidant potential [32]. EMLF7 had the highest sulfate content with negligible levels of protein and polyphenol, indicating that the sulfated polysaccharide was efficiently extracted by the anion exchange column system.

The functional groups were identified using FTIR to elucidate the structure of the sulfated polysaccharide. All of the fucoidan fractions contained a characteristic peak pattern, including the prominent peak observed in commercial fucoidan. The sulfate group (S = O), which is common to all fucoidans, was observed with an intense peak at 1245–1255 cm−1. Further, C-O-S was observed at 825 cm^−1^ and 840 cm^−1^, with similar intensities to those of commercial fucoidan.

The NMR spectra of EMLF7 exhibited the major peaks relevant to polysaccharides and the structure of fucoidan. Among them, the α-anomeric protons and methyl group protons of the sugar residues were prominent. Owing to the structural complexity and heterogeneity of the fucoidans, detailed information regarding the bond formation could not be obtained and will require additional purification procedures, such as de-acetylation and de-sulfation.

Previously published studies on fucoidans isolated from brown algae showed a positive correlation between the degree of sulfation and anti-inflammatory activity [34,53,54]. This was further solidified by the study performed to analyze the effect of the molecular mass and sulfate content of fucoidan on the anti-inflammatory activity. The fucoidan fraction that has a high sulfate content and low molecular weight showed high anti-inflammatory activity [55]. However, further investigations are required for analyzing the correlation between the sulfate and polyphenol contents in the fucoidan fractions in terms of anti-inflammatory activity. The present study selected EMLF7 for further evaluations based on these factors and its superior activity against PM-induced NO production. 

NO is a key inflammatory mediator with diverse physiological and pathological functions. It also plays a major role in pathogenesis [56]. It is released at high concentrations by cytokine-activated macrophages because of iNOS synthesis, which is expressed in cells during a variety of inflammatory diseases. Increased production of NO in the cells leads to tissue damage [57]; thus, NO inhibition is an important therapeutic strategy for combating inflammatory diseases. 

The iNOS and COX-2 expressions are controlled by pro-inflammatory cytokines [58], such as TNF-α, IL-1β, and IL-6, which are implicated in numerous autoimmune and inflammatory diseases, including rheumatoid arthritis, uveitis, and sclerosis [59]. Thus, inhibiting these inflammatory mediators is also essential. However, the IC50 value of fucoidan for the inhibition of these inflammatory mediators can be varied depending on the evaluation method [34,60,61]. According to our study, PM treatment resulted in increased release of NO, PGE2, and pro-inflammatory cytokines, and their production was significantly downregulated by treatment with EMLF7. These results agreed with the results obtained in previously conducted studies [34,53,62].

The inhibitory effect of EMLF7 on the signal transduction of the RAW 264.7 macrophage cells was also determined. The MAPK and NF-κB pathways are important signaling pathways in immune responses. It has been previously reported that PM activates these signaling cascades, resulting in inflammatory responses in the body [20,63,64]. Both MAPK and NF-κB play a vital role in the production of pro-inflammatory mediators. The activation of the NF-κB pathway by PM indicates the phosphorylation translocation of the NF-κB dimers (p65 and p50) to the nucleus, which in turn induces iNOS and COX-2 gene transcription and pro-inflammatory cytokine gene encoding [65]. In agreement with the previous reports, the results of the present study showed that PM stimulation promoted p65 and p50 phosphorylation, while it was downregulated with dose-dependent EMLF7 treatment. Comparable results were obtained in a study conducted on the anti-inflammatory activity of *Sargassum horneri* against PM-induced lung inflammation [7].

MAPK plays a major role in activating chemokines and pro-inflammatory cytokines [66,67]. Among the three important MAPKs (p38, JNK, and ERK), p38 is involved in regulating the synthesis of inflammatory regulators [68,69]. Furthermore, in the pharmacological treatment of inflammatory diseases, ERK, JNK, and p38 are considered important integrators [59]. Several studies have demonstrated the ability of PM to induce the MAPK signaling pathway by the phosphorylation of MAPK-related proteins. Similarly, the results obtained from the present study demonstrated the above-mentioned effect of PM. However, the dose-dependent treatment with EMLF7 downregulated the phosphorylation of MAPK-related proteins and inhibited the activation of the MAPK signaling pathway. These results are in line with results obtained from previous studies conducted on the ability of fucoidan to inhibit inflammatory pathways [7,70].

These results suggest that fucoidan isolated from *E. maxima* is an effective inhibitor of the pro-inflammatory mediators and cytokines through the suppression of the MAPK and NF-κB signaling pathways; therefore, it can be used in the production of anti-inflammatory agents to treat inflammatory diseases.

## 4. Materials and Methods

### 4.1. Materials

Urban PM (CRM No. 28) was purchased from the National Institute for Environmental Studies (Ibaraki, Japan). Potassium bromide (FTIR grade), polysaccharide standards (commercial fucoidan), 3-4,5-dimethylthiazol-2-yl-2,5-diphenyltetrazolium bromide (MTT), 2-propanol, ethanol, the BCA protein assay kit, and chloroform were obtained from Sigma-Aldrich (St. Louis, MO, USA). The murine macrophage cell line RAW 264.7 (ATCC TIB-71) was purchased from the American Type Culture Collection (Rockville, VA, USA). Dulbecco’s modified Eagle’s medium (DMEM), fetal bovine serum (FBS), and penicillin–streptomycin were purchased from Gibco/BRL (Burlington, ON, Canada). Celluclast and Alcalase were purchased from Novo Co. (Novozyme Nordisk, Bagsvaerd, Denmark). The enzyme-linked immunosorbent assay kits for PGE2, IL-6, and IL-1β were acquired from R & D System Inc. (Minneapolis, MN, USA). The primary and secondary antibodies were purchased from Santa Cruz Biotechnology (MP, CA, USA). The enhanced chemiluminescence reagent was obtained from Amersham (Arlington Heights, IL, USA). All other chemicals and solvents were of analytical grade. The commercial fucoidan (Cat. No. F8315) was purchased from Sigma-Aldrich (St. Louis, MO, USA). The biological source of this fucoidan was *Undaria pinnatifida.*

### 4.2. CRM No. 28 Particulate Matter

The certified reference material (CRM) was developed by the National Institute for Environmental Studies of Japan (NIES) to determine the elements in particulate matter. According to the certificate issued by NIES, 99% of them are smaller than 10 µm. The homogeneity of the material was confirmed by the company and the standard deviation was less than 3%. The size distribution results of the PM expressed that the majority of PM has a diameter of around 2 µm. Further, it consists of eight polycyclic aromatic hydrocarbons, and among them, the highest mass fraction was that of benzo (b) fluoranthene. Magnesium, calcium, strontium, barium, and inorganic materials were detected as earth metals, and manganese and lead were detected as transition metals that filled a larger amount of the mass fraction.

### 4.3. Collection and Preparation of the Crude Polysaccharide Sample

*E. maxima* was collected from the coastal area of Cape Town, South Africa, in February 2019. The algae species was identified and kindly provided by Prof. John J. Bolton, University of Cape Town, South Africa. The harvested samples were washed with tap water to remove any impurities, such as salts, epiphytes, and debris, and stored in a −20 °C freezer until further processing. The samples were then dried using a hot-water Goodle dryer and powdered [71]. The powdered samples were depigmented using 95% ethanol solution and suspended in ethanol containing 10% formaldehyde. This was followed by washing the samples with 95% ethanol at room temperature to remove any remaining formaldehyde, pigments, and lipids. The ethanol from the sample was evaporated to total dryness. Then, 100 g of the powder was suspended in 1 L of distilled water and the pH was adjusted to 4.5 by 1M HCL. Celluclast-assisted extraction was initiated using 0.5% celluclast enzyme at 50 °C for 24 h with continuous shaking. After the extraction was complete, the enzyme was heat-inactivated and any debris was removed using centrifugation. The pH in the supernatant was adjusted to 8.0 using NaOH. Then, alcalase-assisted extraction was initiated for the total digestion of the remaining proteins. The alcalase enzyme (Novozyme Nordisk, Bagsvaerd, Denmark) (0.5%) was used for the alcalase-assisted enzymatic extraction of the sample at 50 °C for 24 h in a shaking incubator. Once the extraction was complete, the enzyme was heat-inactivated, the pH of the solution was adjusted to 5.0 by 1M HCl, and a CaCl_2_ solution was added to ensure the precipitation of alginate. Any debris was removed, and the solution was concentrated to one-third of its original volume. Then, the polysaccharides were precipitated by adding three volumes of 95% ethanol. The mixture was kept at 4 °C for 8 h. The precipitated polysaccharides were obtained by centrifugation and homogenized using distilled water. The supernatants were freeze-dried and stored at −20 °C for subsequent experiments [34].

### 4.4. Proximate Composition Analysis

The Association of Official Analytical Chemists (AOAC) method was used to analyze the chemical composition (moisture content, lipid content, protein content, sulfate content, and ash content) of the crude polysaccharide extract of *E. maxima* [59]. The total phenol–sulfuric method was used for measuring the total polysaccharide content of the crude extract and the BaCl_2_–gelatin method was used to determine its sulfate content according to a previously reported method with slight modifications [60]. The protein content was measured using the BCA protein assay kit, the total lipid content was analyzed using the Soxhlet method, and the phenolic content was determined using the folin–ciocaltea method. The polysaccharide, protein, and polyphenol contents were measured at each step of Celluclast extraction and ethanol precipitation of the Celluclast extract accordingly [61,72]. The protein content was measured using the BCA protein assay kit, the total lipid content was analyzed using the Soxhlet method, and the phenolic content was determined using the folin–ciocaltea method.

### 4.5. Separation and Purification by Anion-Exchange Chromatography

The crude extract obtained from *E. maxima* was dissolved in 10 mL of water and passed through a DEAE–cellulose column (30 × 400 nm). The column was equilibrated with sodium acetate (50 mM and pH 5.0) and eluted in a stepwise gradient, starting with 0.2 M NaCl to 2.0 M NaCl, in a buffer system. The final elution was collected and the polysaccharides were quantified using the phenol–sulfuric method and divided into seven fractions: EMLF1–EMLF7. Dialysis was carried out using dialysis membranes to eliminate ionic contaminants.

### 4.6. FTIR Spectroscopy, NMR Analysis, and Monosaccharide Quantification, and Molecular Weight Determination of the Fucoidan Fractions

A FTIR spectrometer (Thermo Scientific Nicolet TM 6700, MA, USA) was used to analyze each fucoidan fraction. The samples were homogenized with KBr powder and pressed into pellets. The FTIR spectra of the fractions were measured in a frequency range of 500–4000 cm^−1^ [60]. The NMR spectrum of fucoidan extracted from *E. maxima* was obtained using a JEOL JNM-ECX400 400MHz spectrometer (Kobe, Japan) at 33 k. The sample preparation and analysis were conducted according to a previously reported method [33,73]. The monosaccharide composition was measured using a previously reported method. Briefly, the fractions were hydrolyzed using 2 M trifluoroacetic acid (TFA) in a sealed glass for 4 h at 100 °C and digested with HCl (6 N) for 4 h. An ED50 Diones electrochemical detector (Dionex, USA) was used to determine the monosaccharide content. The results were analyzed with PeakNet Software (PeakNet IA Software, Waltham, MA, USA) [74].

Agarose gel electrophoresis was utilized to evaluate the homogeneity and purity of the purified polysaccharide fractions using a previously established method with minor modifications [75]. Four molecular weight markers were used to analyze the molecular weight distribution; MW 50-500 kDa (Dextran sulfate, D8906, Sigma), MW ≈ 60 kDa (Chondroitin 6-sulfate, C4384, Sigma), MW ≈ 50 kDa (Dextran sulfate, D8906, Sigma), and MW ≈ 8 kDa (Dextran sulfate, D4911, Sigma). Electrophoresis was performed for 20 min at 100 V using 1% agarose gel with Tris–Borate–EDTA running buffer. The gel was stained and de-stained using 0.02% o-Toluidine in 3% acetic acid containing 0.5% Triton X-100 and 3% acetic acid, respectively [34]. 

### 4.7. Cell Culture

The RAW 264.7 cells were cultured in 10% FBS and 1% penicillin–streptomycin supplemented DMEM. The cells were maintained at 37 °C in a 5% CO_2_ humidified atmosphere until further analysis.

### 4.8. Cell Viability and NO Production

An MTT test was conducted to assess the cell viability of the PM-stimulated cells. The RAW 264.7 macrophages were seeded (1 × 10^5^ cells/mL) in a 24-well plate and incubated at 37 °C for 24 h. Then, the cells were treated with the fucoidan extracts (25, 50, 100, and 200 μg/mL), incubated for 2 h, and then stimulated with PM (125 μg/mL) for 24 h. The protective effect of fucoidan against the PM-stimulated cells was measured using the MTT assay.

To determine the extent of NO production in the PM-stimulated cells, first, the RAW 264.7 cells were seeded at the above-mentioned concentration and incubated for 24 h at 37 °C in a 5% CO_2_ humidified atmosphere. Then, the cells were treated with different concentrations of the fucoidan extracts (25, 50, 100, and 200 μg/mL) and further incubated for 2 h, followed by PM stimulation (125 μg/mL). Finally, equal amounts of the culture medium and Griess reagent were reacted in a 96-well plate for 10 min, and the absorbance was measured at 540 nm using an ELISA plate reader (BioTek Instruments, Inc, Winooski, VT, USA) [61]. Based on the superior anti-inflammatory activity demonstrated by the EMLF7 fraction, it was used for the subsequent experiments.

### 4.9. Determination of PGE2 and Pro-Inflammatory Cytokine Production

Prostaglandins play a vital role in regulating inflammatory responses. Therefore, PGE2 production in PM-stimulated RAW 264.7 macrophage cells was measured using an ELISA kit according to the manufacturer’s instructions. The cultured cells were treated with the measured concentrations of the EMLF7 fraction, incubated for 1 h, stimulated with PM (125 μg/mL), and incubated for a further 24 h. Then, the concentrations of PGE2, TNF-α, IL-6, and IL-1β in the supernatant were quantified using competitive enzyme immunoassay kits (R & D System Inc., Minneapolis, MN, USA). Dexamethasone (50 μM) was used as a reference in this study.

### 4.10. Western Blot Analysis

To determine the effect of fucoidan on the protein expression levels of iNOS, COX-2, NF-κB, and MAPK in the PM-stimulated RAW 264.7 cells, Western blot analysis was performed. Briefly, cells were seeded at a 1 × 10^5^ cells/mL concentration in a six-well plate, incubated for 24 h, treated with the EMLF7, incubated for 1 h, stimulated with PM (125 μg/mL), and further incubated for 24 h. Then, the cells were harvested, and nucleic and cytosolic proteins were extracted from the cells using a NE-PER@ Nuclear and Cytoplasmic extraction kit (Thermo Scientific, Rockford, IL, USA). The protein levels were evaluated using a BCA protein assay kit. The proteins were subjected to sodium dodecyl sulphate–polyacrylamide gel electrophoresis (SDS-PAGE) (12%) and the transfer was completed on a nitrocellulose membrane. Subsequently, the membranes were blocked with 5% skimmed milk for 2 h, and the blocked membranes were incubated with primary antibodies overnight. Thereafter, horseradish peroxidase (HRP)-conjugated secondary antibodies were added to the membrane and incubated at room temperature for 2 h. Signals were developed using a chemiluminescent substrate (Cyanagen Srl, Bologna, Italy) and photographed via a FUSION SOLO Vilber Lourmat system (Paris, France). Finally, the band intensities were quantified using the ImageJ program [76].

### 4.11. RNA Extraction and cDNA Synthesis

The total RNA from the *E. maxima* fucoidan-treated RAW 264.7 cells was extracted using a Tri-ReagentTM extraction kit (Sigma Aldrich, St. Louis, MO, USA). The RNA samples were transcribed using a cDNA Reverse Transcription Kit (Takara, Shiga, Japan). RT-qPCR amplification reactions of cDNA were conducted using a Thermal Cycler Dice Real-Time System (Takara, Japan) in the following manner: enzyme activation at 95 °C for 10 s, followed by 40 cycles of denaturation at 95 °C for 5 s, and annealing at 58 °C for 10 s. The reaction was carried out in a 10 μL volume containing 3 μL of cDNA, 5 μL of the 2x TaKaRa ExTaq SYBR premix (TaKaRa, BIO INC, Japan), 0.4 μL of each primer, and 12 μL of RNase/DNase-free water. Using GAPDH as an internal reference standard gene in the amplification process, the pro-inflammatory cytokine expression levels were evaluated. The primers used in the experiment are listed below [61].

GAPDH, forward; 5′-AAGGGTCATCATCTCTGCCC-3′ and reverse, 5′-GTGATGGCATGGACTGTGGT -3′,

iNOS, forward; 5′-ATGTCCGAAGCAAACATCAC-3′ and reverse, 5′-TAATGTCCAGGAAGTAGGTG-3′, COX2, forward; 5′-CAGCAAATCCTTGCTGTTCC -3′ and reverse, 5′-TGGGCAAAGAATGCAAACATC-3′,

IL-1β, forward; 5′-CAGGATGAGGACATGAGCACC-3′ and reverse, 5′- CTCTGCAGACTCAAACTCCAC -3′,

IL-6, forward; 5′-GTACTCCAGAAGACCAGAGG -3′ and reverse, 5′-TGCTGGTGACAACCACGGCC-3′,

TNF-α, forward; 5′-TTGACCTCAGCGCTGAGTTG -3′ and reverse, 5′- CCTGTAGCCCACGTCGTAGC -3′,

TLR2, forward; 5′-CAGCTGGAGAACTCTGACCC-3′ and reverse, 5′- CAAAGAGCCTGAAGTGGGAG-3′, and

TLR4, forward; 5′- CAACATCATCCAGGAAGGC -3′ and reverse, 5′- GAAGGCGATACAATTCCACC -3′.

### 4.12. Statistical Analysis

All the data are represented as the mean ± standard deviation of three measurements. The mean values were compared using one–way analysis of variance. Duncan’s multiple range test was used for the mean separation. *P*-values of < 0.05 as * and *p*-values of < 0.01 as ** were considered statistically significant.

## 5. Conclusions

In conclusion, these results show that fucoidan from *E. maxima* can be used to inhibit PM-induced inflammation-driven pathways. Fucoidan significantly suppressed the production of NO, PGE2, and pro-inflammatory cytokines, such as TNF-α, IL-6, and IL-1β, by inhibiting NF-κB and MAPK activation in the PM-stimulated RAW 264.7 cells. Therefore, leaf fucoidan isolated from the brown algae *E. maxima* has the potential to be used as an anti-inflammatory agent against inflammatory diseases. In addition, owing to its simple extraction process and abundance, it can be used as an inexpensive source of fucoidan.

## Figures and Tables

**Table 1:** Chemical composition of fucoidan fractions from *E. maxima* leaves.

	Polysaccharide Content %	Sulfate Content %	Protein Content %	Polyphenol Content %	Sulfated Polysaccharide Content %
Crude fucoidan	60.54	23.63	2.82	2.01	84.17
*EMLF1*	76.53	11.98	1.41	1.08	88.51
*EMLF2*	72.38	18.69	0.86	1.07	91.07
*EMLF3*	69.55	22.64	0.86	0.95	92.19
*EMLF4*	65.49	25.16	0.61	0.74	90.65
*EMLF5*	60.14	32.67	0.55	0.64	92.81
*EMLF6*	56.89	35.11	0.53	0.47	92
*EMLF7*	51.44	39.76	0.48	0.32	91.2

*E. maxima* purified leaf fucoidan fraction (EMLF).

**Table 2:** Monosugar composition of *E. maxima* leaf fucoidan fractions.

	*EMLF1*	*EMLF2*	*EMLF3*	*EMLF4*	*EMLF5*	*EMLF6*	*EMLF7*
Fucose	0.73	16.46	28.82	33.01	36.83	56.68	81.83
Rhamnose	ND	1.34	0.96	0.86	1.86	1.08	0.33
Arabinose	ND	0.66	4.1	0.14	0.23	0.12	0.04
Galactose	ND	11.51	20.42	18.7	34.17	25.01	14.67
Glucose	99.27	46.27	4.31	5.76	ND	ND	ND
Mannose	ND	23.76	41.38	41.5	26.91	17.11	3.13
Yield %	9.55 ± 0.56	9.04 ± 0.22	10.74 ±0.42	11.32 ± 0.26	9.51 ± 0.62	9.36 ± 0.49	9.11 ± 0.68

*E. maxima* purified leaf fucoidan fraction (EMLF).

## Supplementary Materials

The following supporting information can be downloaded at: https://www.mdpi.com/article/10.3390/md20070413/s1, Figure S1: Molecular weight analysis of the purified fucoidan fractions.
