# Supplementary material for: Anti-Fine Dust Effect of Fucoidan Extracted from Ecklonia maxima Laves in Macrophages via Inhibiting Inflammatory Signaling Pathways
Source: Mar Drugs. 2022 Jun 24;20(7):413. doi: 10.3390/md20070413 (PMC9319110; doi:10.3390/md20070413)
Supplement: Supplementary file 1 [file marinedrugs-20-00413-s001.zip › marinedrugs-1755387-supplementary.pdf]

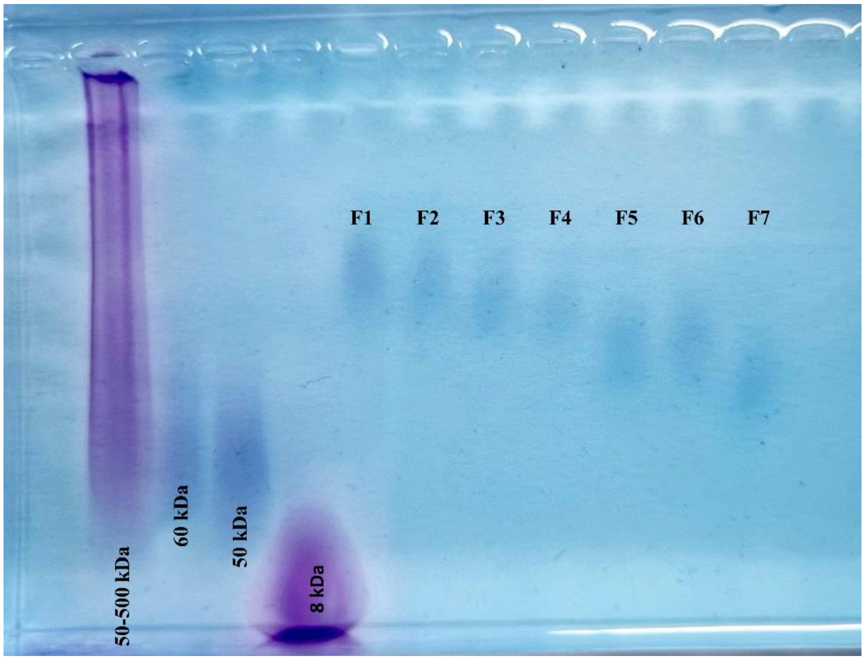

Figure S1. Molecular weight analysis of purified fucoidan fractions. Molecular weight distribution of the seven fractions were evaluated using agarose gel electrophoresis with comparison of polysaccharide standard with defined molecular weights. MW 50-500 kDa (Dextran sulfate, D8906, Sigma), MW ≈ 60 kDa (Chondroitin 6-sulfate, C4384, Sigma), MW ≈ 50 kDa (Dextran sulfate, D8906, Sigma), and MW ≈ 8 kDa (Dextran sulfate, D4911, Sigma). Fraction 1-7 (F1-F7)
